# Inflammatory Manifestations of Experimental Lymphatic Insufficiency

**DOI:** 10.1371/journal.pmed.0030254

**Published:** 2006-07-18

**Authors:** Raymond Tabibiazar, Lauren Cheung, Jennifer Han, Jeffrey Swanson, Andreas Beilhack, Andrew An, Soheil S Dadras, Ned Rockson, Smita Joshi, Roger Wagner, Stanley G Rockson

**Affiliations:** 1Stanford Center for Lymphatic and Venous Disorders, Division of Cardiovascular Medicine, Stanford University School of Medicine, Stanford, California, United States of America; **2**Department of Pathology, Stanford University School of Medicine, Stanford, California, United States of America; University College LondonUnited Kingdom

## Abstract

**Background:**

Sustained lymph stagnation engenders a pathological response that is complex and not well characterized. Tissue inflammation in lymphedema may reflect either an active or passive consequence of impaired immune traffic.

**Methods and Findings:**

We studied an experimental model of acute post-surgical lymphedema in the tails of female hairless, immunocompetent SKH-1 mice. We performed in vivo imaging of impaired immune traffic in experimental, murine acquired lymphatic insufficiency. We demonstrated impaired mobilization of immunocompetent cells from the lymphedematous region. These findings correlated with histopathological alterations and large-scale transcriptional profiling results. We found intense inflammatory changes in the dermis and the subdermis. The molecular pattern in the RNA extracted from the whole tissue was dominated by the upregulation of genes related to acute inflammation, immune response, complement activation, wound healing, fibrosis, and oxidative stress response.

**Conclusions:**

We have characterized a mouse model of acute, acquired lymphedema using in vivo functional imaging and histopathological correlation. The model closely simulates the volume response, histopathology, and lymphoscintigraphic characteristics of human acquired lymphedema, and the response is accompanied by an increase in the number and size of microlymphatic structures in the lymphedematous cutaneous tissues. Molecular characterization through clustering of genes with known functions provides insights into processes and signaling pathways that compose the acute tissue response to lymph stagnation. Further study of genes identified through this effort will continue to elucidate the molecular mechanisms and lead to potential therapeutic strategies for lymphatic vascular insufficiency.

## Editors' Summary

**Background.:**

Lymphedema is the term used to describe the swelling that can occur after surgery, especially after axillary lymph node dissection for breast cancer, when the lymph vessels that carry protein-rich interstitial fluid from the tissues to the heart are damaged. Lymphedema can be extremely unpleasant and is very hard to treat; treatments that are currently used include those aimed to help massage the flow of lymph back to the chest. Lymphedema seems to be more than just accumulation of lymph, however, as changes also occur in the tissue surrounding the damaged lymph vessels. Currently, very little is known at the most basic level about what exactly these changes are, although they appear to be similar to an inflammatory process. One way of studying such a disease process in humans is to make an animal model that mimics the human condition and study the changes that occur there. For lymphedema, such a model can be made in the mouse tail by cutting lymph vessels there.

**Why Was This Study Done?:**

The authors wanted to look closely at what happens in the tissues surrounding damaged lymph vessels to try to understand better what these changes are. They also wanted to study the movement of the cells that are normally carried in the lymph.

**What Did the Researchers Do and Find?:**

The mouse model that the authors developed closely simulates the characteristics of human acquired lymphedema. In the mouse tails that had had their lymph vessels damaged, the authors were able to show that the tails were swollen compared with those of normal animals and of animals that hadhad sham (pretend) surgery. In the animals with lymphedema, many small lymph vessels were seen, as the lymph was unable to flow away normally. The area affected by the lymphedema had the appearance often seen in inflamed tissue, and analysis of genes from the same area to see how active, or “expressed,” they were showed changes that are often seen in, for example, acute inflammation and wound healing. The authors also showed that when these animals were injected with immune cells marked with a light marker, they were less able to remove the cells from the circulation.

**What Do These Findings Mean?:**

These results show that the response to lymph stagnation is complex, but looks similar to that seen in acute inflammation. These results and this model may be useful in suggesting, and at a later date perhaps testing, treatments for lymphedema. One difference, however, between this mouse model and the condition in humans is that whereas lymphedema in humans is a rather chronic condition, here the researchers were only able to look at the changes over a short period of time. In a related Perspective article, Peter Carmeliet and colleagues further discuss the clinical relevance of these findings ( http://dx.doi.org/10.1371/journal.pmed.0030264).

**Additional Information.:**

Please access these Web sites via the online version of this summary at http://dx.doi.org/10.1371/journal.pmed.0030254.

• The National Cancer Institute has information for patients and health professionals on lymphedema

• Cancerbackup is a United Kingdom cancer information service with information on many aspects of cancer, including lymphedema

• The Lymphatic Research Foundation Description has information on lymphatic system research

## Abstract 3

Rockson and colleagues characterized a mouse model of acute, acquired lymphedema using in vivo functional imaging, histopathological examination, and transcriptional profiling.

## Introduction

Acquired lymphedema is a common, important, and often devastating consequence of successful surgical and adjuvant therapy of breast cancer and other malignancies [ 1, 2]. The biology of regional lymphatic vascular insufficiency (lymphedema) is complex and, as yet, poorly understood. Consequently, there is a paucity of effective treatment strategies in patients with lymphedema [ 2]. There is an obvious need for better molecular characterization of this disease process to elucidate the pathobiology of lymphatic vascular insufficiency.

The tissue response to lymph stagnation is rather complex. The profound structural and functional abnormalities in the lymphedematous tissues reflect a multicellular response to impaired extracellular fluid mobilization [ 3]. It has been suggested that lymphedema provokes an inflammatory tissue response in the skin [ 4]. While it is conceivable that the inflammatory nature of the tissue response to lymph stagnation reflects either the active or passive consequences of impaired immune traffic [ 5], direct experimental confirmation is lacking.

Transcriptional profiling has been utilized in the molecular characterization of isolated lymphatic endothelia [ 6, 7], but the molecular end-organ response to lymph stagnation remains unaddressed and poorly understood. While lymphatic and blood vessel responses to injury might be predictable, an elucidation of whole tissue response to disease is likely to provide more relevant insights into the important interactions between the tissue matrix and the resident, heterogeneous cellular populations that likely compose the target-organ response to persistent lymph stagnation.

To investigate tissue responses to lymphatic vascular insufficiency, we have undertaken dynamic, in vivo imaging of the impaired immune traffic in a murine model of acquired lymphatic insufficiency that is intended to simulate, in part, the lymphatic dysfunction of post-surgical lymphedema [ 8]. These observations were correlated with an assessment of the cutaneous histopathology in the lymphedema tissue. Furthermore, to investigate the molecular mechanisms of tissue response to lymphatic vascular insufficiency, we have undertaken a large-scale transcriptional profiling of the lymphedema tissue utilizing a comprehensive mouse cDNA microarray containing 42,300 features, representing over 25,000 unique genes and expressed sequence tags (ESTs) [ 9]. The patterns of gene expression in lymphedema were contrasted with those observed in normal and surgical sham controls.

## Methods

This study was approved by the Administrative Panels on Laboratory Animal Care of Stanford University.

### Creation of Experimental Lymphedema

Post-surgical lymphedema was experimentally created in the tails of female hairless, immunocompetent SKH-1 mice (Charles River Laboratories, Boston, Massachusetts, United States). Prior to surgery, the mice were anesthetized with intraperitoneal injection of 0.07 cc of a solution containing ketamine, xylazine, and saline. For each intervention, the skin of the tail was circumferentially incised proximally, at a point 16 mm distal to its base. The major lymphatic trunks were identified through subcutaneous injection of methylene blue distal to the surgical incision, followed by controlled, limited cautery ablation of these structures. In surgical controls (sham animals), skin incision alone was performed, with methylene blue injection but without lymphatic cautery. The normal control animals did not undergo any surgical manipulation. All animal subjects were sacrificed on day 14 of observation. After sacrifice, 0.5-gm sections of the tail were harvested for paraffin embedding and RNA extraction.

### Tail Volume Quantitation

Tail volume was quantitated in each animal subject immediately prior to sacrifice. Volumetric assessment was performed with a manually adjusted caliper, with serial measurement of the tail circumference at 5-mm intervals along its axis. The tail volume was quantitated with the truncated cone formula [ 10].

### Histology

Immediately following sacrifice, 0.5-gm sections of the tail were harvested for histological analysis and RNA extraction. Sections extended from a point 4 mm proximal to the surgical incision to 8 mm beyond it. For examination of the responses remote from the point of injury, sections were harvested 4 cm distal to the surgical site. The specimens were fixed overnight in 4% paraformaldehyde. After paraffin embedding, 5-μm sections were stained with hematoxylin and eosin (Richard-Allan Scientific, Kalamazoo, Michigan, United States). For visualization of histiocytes/mast cells, the sections were stained with a 1% toluidine blue solution (LabChem, Pittsburgh, Pennsylvania, United States) diluted in 1% NaCl. After deparaffinization in xylene, sections were rehydrated though a series of graded alcohol steps starting with 100% EtOH and ending in 50% EtOH. Slides remained in toluidine blue for 2 min and were then dehydrated through graded alcohol washes and covered with Cytoseal (Richard-Allan Scientific).

### LYVE-1 Immunohistochemical Staining

Paraffin sections 5 μm thick were deparaffinized in xylene, rehydrated in a graded series of ethanol, pretreated with target retrieval solution (Dako, Carpinteria, California, United States) in a pressure cooker, and incubated in a peroxidase block for 10 min. Sections were then incubated with rabbit polyclonal anti-LYVE-1 antibody (1:200, Upstate Cell Signaling Solutions, Lake Placid, New York, United States) for 1 h at room temperature, followed by horseradish-peroxidase-conjugated secondary antibody for 30 min at room temperature and detection with DAB for 4 min (Envision System Kit, Dako). Tissue sections were counterstained with Gill 1 hematoxylin (Richard-Allan Scientific) for 15 s, then dehydrated in graded ethanol and coverslipped with CoverSafe (American Master*Tech Scientific, Lodi, California, United States).

### Functional Imaging of Immune Traffic in the Lymphedema Model

Experimental lymphedema was created surgically in the tails of FVB/N female wild-type mice (Jackson Laboratory, Bar Harbor, Maine, United States; *n =* 3), using the technique described above. Surgical sham controls ( *n =* 5) were also created and compared with normal mice ( *n =* 5). For in vivo bioluminescence imaging, spleens from transgenic luciferase ( *luc* ^+^) heterozygous animals were put into single-cell suspension, expressing firefly *luc* under the control of a chicken beta-actin promoter as previously described [ 11, 12]. The single-cell suspensions from mouse spleens consisted of different hematopoietic lineages: ˜40% were CD19+ B cells, ˜20% were CD4+ T cells, ˜10%–15% were CD8+ T cells, 3% were NK1.1+ NK cells, and the rest were GR.1+ granulocytes, Mac-1+ macrophages, CD11c+ dendritic cells, and rarer cell populations. A total of 4 × 10 ^6^ splenocytes (>97% CD45+) in PBS were injected in a volume of 20 ml into the tail interstitium, 1 cm caudal to the site of surgery, in both lymphedema mice and surgical shams. Normal mice were injected at the corresponding level of the tail. Injections were performed on post-surgical day 7. Thereafter, *luc ^+^* cells were repetitively imaged in vivo *,* at predetermined intervals following the cell injections. In brief, mice were anesthetized by intraperitoneal co-injection of a mixture of ketamine (1 mg/mouse), xylazine (μg/mouse) in PBS, and the substrate luciferin (150 mg/kg).

Ten minutes thereafter, dorsal images were obtained with an IVIS100 CCD imaging system (Xenogen, Alameda, California, United States). The efficiency of cellular lymphatic drainage was determined by direct imaging of light emission at each of the measured time points, with quantitation of the change in light emission relative to that observed 20 h after cell injection, which was defined as 100%.

### Microsphere Quantitation of Arterial Perfusion of the Mouse Tail

The arterial perfusion of the tails of experimental and control mice was quantitated through intracardiac microsphere injection. After induction of general anesthesia, stable-labeled 15-μm microspheres (STERIspheres Gold, BioPAL, Worcester, Massachusetts, United States) were injected into the left ventricle. Each animal subject received 0.5 × 10 ^6^ microspheres (0.2 ml) injected directly into the left ventricle. The animals were sacrificed after 12 min. The tails were harvested and dried overnight at 70 °C. The assay to quantitate disintegrations per minute (dpm) was performed by BioPhysics Assay Laboratory (Worcester, Massachusetts, United States) as previously described [ 13].

### Lymphoscintigraphy in Experimental Lymphedema

Whole body lymphoscintigraphy was performed after the intradermal injection of 100 μCi/0.02 ml of filtered ^99m^Tc-sulfur colloid (100 nm size) into the tip of the tail. Dynamic and static images (255 × 255) were acquired using a parallel hole collimator in a microSPECT gamma camera (Lumigem, Gamma Medica, Northridge, California, United States). The dynamic images (1,000 frames; 0.5 s/frame) were started 60 s prior to the injection of the tracer. The injection lasted for 20 s. The static images (10 min) were acquired immediately after the dynamic acquisition.

### Microarray Experimental Design, RNA Preparation, and Hybridization

Tissues were derived from nine mice for each of the three biological states under study (cutaneous specimens from normal, lymphedematous, and surgical sham animals), for a total of 27 mice. All microarray hybridizations were performed with three biological replicates, using pooled samples independently derived from three mice each, for a total of nine hybridizations. After tissue was harvested for histological examination, the remaining, distal portion of the tail was retrieved for RNA isolation. After completely separating the tail skin from the cartilage by blunt dissection, the tissue was separated into segments of 0.5 mm for further processing. Total RNA was isolated using a modified two-step purification protocol as described previously [ 14]. RNA integrity was assessed using the Agilent 2100 Bioanalyzer System with RNA 6000 Pico LabChip Kit (Agilent, Palo Alto, California, United States). First-strand cDNA was synthesized from 15 μg of total RNA derived from each pool and from whole embryonic-day-17.5 embryo for reference RNA, in the presence of Cy3 and Cy5 dUTP, respectively, and hybridized to the Mouse Transcriptome Microarray [ 14– 16]. A continuously updated and annotated list of the cDNAs included on this array is available at the Stanford Microarray Database [ 17] ( Table S1).

### Data Acquisition, Analysis, and Statistical Analysis

Image acquisition of the mouse cDNA microarrays was performed on an Agilent G2565AA Microarray Scanner System. Feature extraction was performed with GenePix 4.0 software (Bucher Biotec, Basel, Switzerland). Numerical raw data were migrated from GenePix, without processing, into an Oracle relational database (CoBi) that was designed specifically for microarray data analysis (GeneData, Basel, Switzerland). The data were then analyzed using Expressionist software (GeneData). After background subtraction and dye normalization, features with low signal intensity in the reference channel were filtered if signal was less than 2.5× background value, retaining a total of 8,353 features for further analysis. *K*-nearest-neighbor algorithm was applied to impute for missing values (<7% of remaining data) [ 18]. For two-group comparisons, we used the significance analysis of microarrays (SAM) algorithm [ 19, 20]. Heat maps were generated using HeatMap Builder [ 21, 22]. For enrichment analysis we used the EASE analysis software, which uses Gene Ontology (GO) annotation and Fisher's exact test to derive biological themes within particular gene sets [ 23].

### Quantitative Real-Time RT-PCR

Quantitative real-time RT-PCR (qRT-PCR) was performed as described [ 14]. Primers and probes for ten representative differentially expressed genes were obtained from Applied Biosystems Assays-on-Demand (Applied Biosystems, Foster City, California, United States. cDNA was synthesized from 5 μg of total RNA using Taqman Reverse Transcription Reagents (Applied Biosystems), a set which includes MultiScribe reverse transcriptase, RNase inhibitor, dNTP mixture, oligo d(T) _16_, random hexamers, 10× RT buffer, and MgCl _2_ solution. Amplification was performed in triplicate at 50 °C for 2 min and 95 °C for 10 min, followed by 40 cycles of 95 °C for 15 s and 60 °C for 1 min. Reactions without template and/or enzyme were used as negative controls. 18S ribosomal RNA was used as an internal control. A standard curve derived from embryonic-day-17.5 mouse RNA was plotted for each target gene by linear regression using SPSS version 11.0 software (Applied Biosystems). RNA quantity was expressed relative to the corresponding 18S control. Fold differences were calculated by dividing the experimental results by the pooled normal results and were plotted on a log10 scale. The primers and probes utilized in this study are listed in Tables 1 and 2.

**Table 1:**
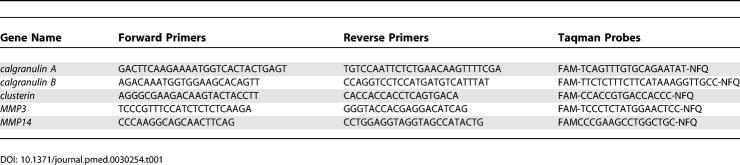
Primer/Probe Sequences for the Taqman-Based qRT-PCR

**Table 2:**
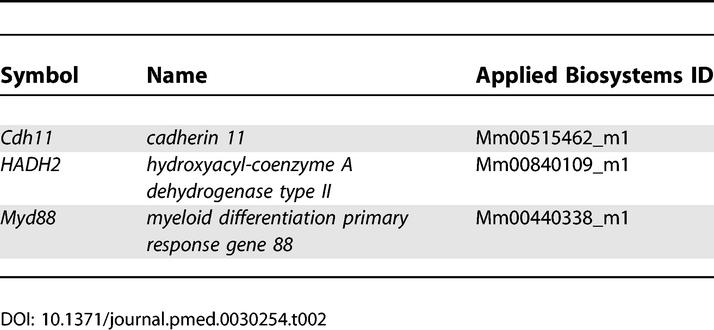
Names of Taqman-Based qRT-PCR Probes

## Results

### Murine Model of Acute Experimental Lymphedema: Tail Volume Quantification

**Figure 1:**
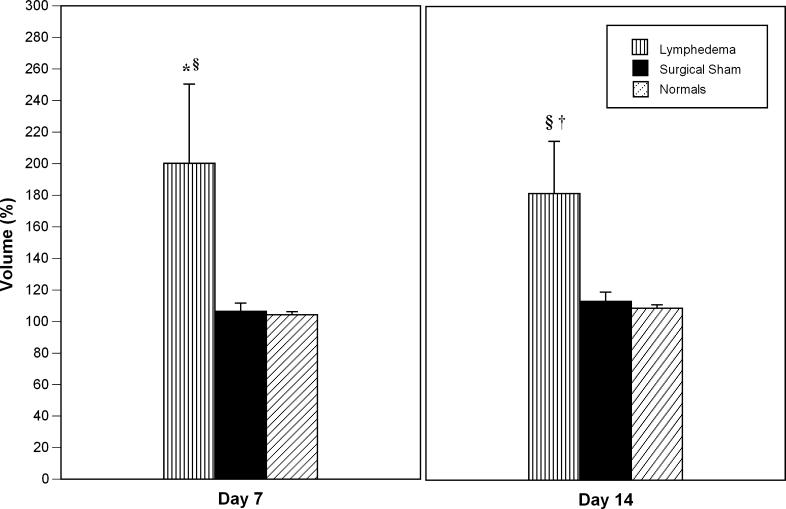
Tail Volume Changes at Post-Surgical Day 7 and at Day 14

Forty-five 3-wk-old SKH-1 hairless mice were studied in this investigation. Of these, 18 underwent post-surgical lymphatic ablation, nine served as surgical sham controls, and the remaining 18 served as normal controls. Tail volume for each group of animals is depicted in Figure 1. At post-surgical day 7, the lymphedema tail volumes were 200% ± 50% of baseline ( *p <* 0.008 when compared to surgical sham controls). In the animals subjected to lymphatic ablation, the edematous enlargement of the tails persisted until the day of sacrifice (day 14). Of note is the fact that cutaneous healing of the wound, both in the lymphedematous and surgical sham subjects, was complete by day 14. There was no statistically significant change in tail volume in either surgical sham or normal controls.

### Histological Assessment of the Cutaneous Response to Lymphatic Interruption

**Figure 2:**
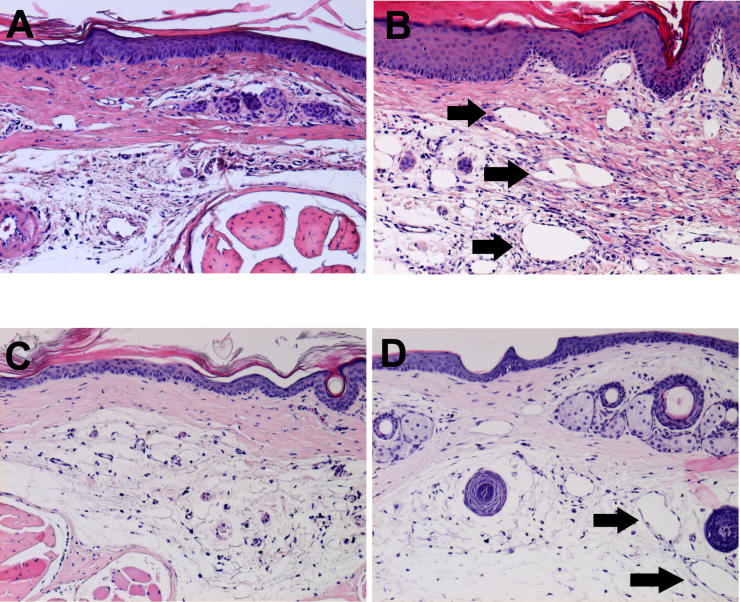
Histopathology of Experimental Lymphedema in the Murine Tail Lymphedema was characterized by the presence of marked acute inflammatory changes, both adjacent to the surgical site and within distal regions of the tail, remote from the site of surgical ablation. (A) Normal tail skin harvested 16 mm from the base of the tail is characterized by the presence of a thin dermis and epidermis, with a normal epidermal/dermal junction. Surgical sham controls were indistinguishable from normals, with no increased cellularity in dermis or epidermis, and no enlarged nuclei or hyperkeratosis. (B) Lymphedematous skin harvested immediately distal to the site of prior surgical lymphatic ablation is characterized by the presence of marked acute inflammatory changes, absent in the tissue derived from the normal tails. There is a notable increase in cellularity, with an increase in the number of observed fibroblasts and histiocytes, as well as a large infiltration of neutrophils. There is hyperkeratosis and spongiosis and edema of the epidermis, with irregularity of the epidermal/dermal junction, elongation of the dermal papillae, and a 2- to 3-fold expansion of tissue between the bone and the epidermis. There are numerous dilated lymphatic microvessels in the dermis and subdermis (black arrows). In contrast, normal tail sections were devoid of these dilated structures. (C) Normal skin derived from the distal tail. No inflammation, hypercellularity, or lymphatic dilatation is observed. (D) Distal skin in lymphedema. Spongiosis and lymphatic microvascular dilatation (black arrows) are once again detectable.

Hematoxylin and eosin specimens derived from the lymphedematous tails were characterized by the presence of marked acute inflammatory changes ( Figure 2B), when compared to the tissue derived from the normal tails ( Figure 2A). There was a notable increase in cellularity, with an increase in the number of observed fibroblasts and histiocytes, as well as a large infiltration of neutrophils Granulation tissue was observed closer to the center of the section, with bystander destruction of muscle tissue. In addition, there was hyperkeratosis and spongiosis and edema of the epidermis, with irregularity of the epidermal/dermal junction, elongation of the dermal papillae, and a 2- to 3-fold expansion of tissue between the bone and the epidermis. Lymphedema specimens were characterized by the presence of numerous dilated lymphatics in the dermis and subdermis, as seen in Figure 2B. In contrast, normal tail sections were devoid of these dilated structures. The normal tissues were characterized by the presence of a thin dermis and epidermis, with a normal epidermal/dermal junction ( Figure 2A). The surgical sham controls were indistinguishable from normals, with no increased cellularity in dermis or epidermis, and no enlarged nuclei or hyperkeratosis.

In order to assess whether the lymphedematous changes created a uniform pathological response distal to the point of lymphatic ablation, the tissues were also sampled distally (4 cm distal to the point of surgical incision) in normal ( Figure 2C) and lymphedematous ( Figure 2D) tails. The observed changes were comparable to those observed adjacent to the surgical site: lymphedematous tissues were characterized by hypercellularity, inflammatory infiltration, and microlymphatic dilatation that were not present in the normal tissues.

### Quantitative Assessment of Arterial Perfusion in the Murine Tail Lymphedema Model

While we took great care to avoid concurrent injury to adjacent vascular structures during surgical lymphatic ablation, we have undertaken an evaluation to exclude inadvertent arterial injury during surgery. The mouse tails remained grossly stable throughout the post-surgical observation phase, with no evidence of frank necrosis distal to the surgical site. In order to further substantiate the absence of an arterial ischemic contribution to the histological pathology observed in lymphedema, quantitative assessment of arterial perfusion was performed through intracardiac injection of stable 15-μm microspheres into the left ventricles of normal ( *n =* 3) and lymphedema ( *n =* 3) mice. Perfusion of the tail, measured in disintegrations per minute (dpm), did not differ statistically between the two categories (normal, 151,186 ± 69,213 dpm; sham, 95,581 ± 48,003 dpm), confirming preservation of arterial supply in the lymphedema animals.

### LYVE-1 Immunohistochemical Staining

**Figure 3:**
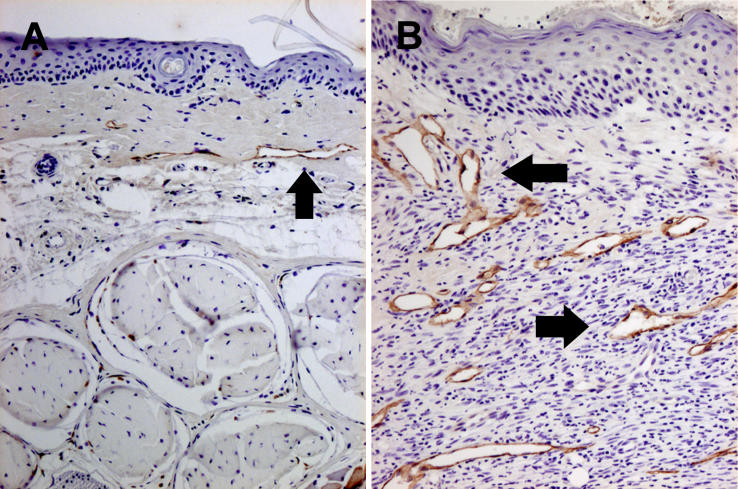
LYVE-1 Immunohistochemical Staining Immunohistochemical staining for LYVE-1 is depicted in surgical sham controls (A) and in lymphedema (B) (black arrows). The lymphedema response is characterized by the presence of numerous dilated microlymphatic structures in the dermis and subdermis. Lymphedema produces a statistically significant increase in average cross-sectional vessel area.

The nature of the lymphatic vascular response distal to the anatomic surgical ablation was assessed with quantitative assessment of lymphatic vessel number and size by immunohistochemical staining for LYVE-1( Figure 3) [ 24, 25]. As observed in the hematoxylin and eosin sections, lymphedema was characterized by the presence of numerous dilated microlymphatic structures in the dermis and subdermis. Mean lymphatic vessel number was determined by averaging the number of total lymphatic vessels in all the fields of each slide at 10× magnification. Single brown-stained endothelial cells with a lumen were counted as individual lymphatic vessels. Quantitation was performed for normals ( *n =* 3), surgical shams ( *n =* 3), and lymphedema tails ( *n =* 3). Lymphedema was characterized by an increase in LYVE-1-positive vessel number per field that was not observed in shams: lymphedema, 7.0 ± 4.8; sham, 0.6 ± 0.5; and normal, 1.2 ± 0.8.

Vessel area was quantitated according to the formula π· *r*_1_· *r*_2_. The average lymphatic luminal area per field was 503 ± 158 μm ^2^ in normals, 436 ± 345 μm ^2^ in shams, and 51,344 ± 18,688 μm ^2^ in lymphedema. Normals and shams did not differ statistically, but the lymphedema group displayed a statistically significant increase in average vessel area when compared either to normals or to sham surgical animals ( *p* = 0.009 for each comparison). Thus, in summary, the experimental lymphedema is accompanied by an increase in vessel number and, even more notably, by an increase in lymphatic vascular cross-sectional area.

### Lymphoscintigraphy of Experimental Lymphedema

Whole body lymphoscintigraphy was performed in normal ( *n =* 4) and lymphedema ( *n =* 4) mice. All non-operated mice showed lymphatic drainage from the tip of the tail through two lumbar lymph nodes, asymmetric para-aortic nodes, and mediastinal nodes with final visualization of the liver. The lymphatic flow speed in basal conditions was estimated to be 0.9 ± 0.66 mm/s. In the lymphedema animals, significant dermal backflow was present, but no flow was observed beyond the base of the tail. These lymphoscintigraphic findings closely simulate the qualitative changes observed in the analogous imaging of acquired human lymphedema.

### Functional In Vivo Imaging of Immune Traffic

The lymphatic vasculature participates in the immune response through the continuous transportation of white blood cells and antigen-presenting cells. The constellation of histological observations in this model, otherwise unexplained by impaired interstitial fluid mobilization, suggests that derangements in lymphatic immune traffic might contribute—actively, passively, or both ways—to the biology of lymph stagnation. Accordingly, we chose to corroborate histopathology with observed, quantifiable changes in immune traffic.

**Figure 4:**
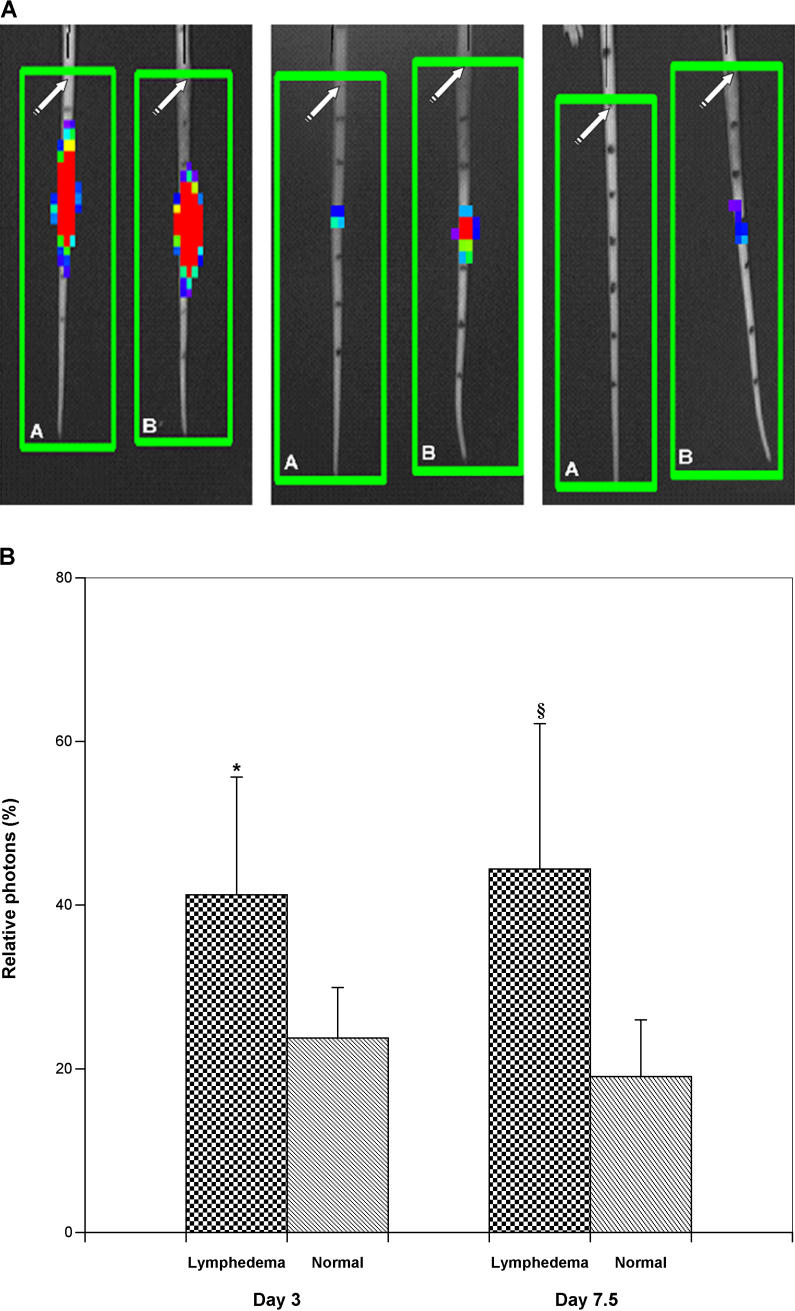
Dynamic Imaging of Immune Traffic in Experimental Lymphedema (A) In vivo bioluminescence imaging of immune traffic. Bioluminescence imaging was performed at defined time points following the introduction of *luc ^+^* cells. This figure contains a representative series of imaging experiments for paired normal control (A) and lymphedema (B) mice. Photon densities range from red (high) to blue (low). In general, clearance of bioluminescent immunocytes from the lymphedematous tails was delayed, but remained unimpaired in the surgical sham controls. The left panel shows a perceptible increase in photon densities in lymphedema on day 3 post-injection (post-operative day 10). Within several days, the disparity in cellular clearance is even more evident (middle panel); as late as day 17 post-injection, there is still visible bioluminescence in the lymphedematous tail, while all activity has cleared from the normal tail (right panel). The original surgical site is depicted by the white arrows. The black marks on the tail denote 8-mm vertical distances; splenocyte injection was performed 24 mm below the surgical site. (B) Quantitative assessment of in vivo bioluminescence imaging of immune traffic. Relative photon density, expressed as a percent of the observed value on day 1, was significantly greater in lymphedema than in normal controls, both at day 3 and at day 7 post-injection (*, *p <* 0.05; §, *p <* 0.02).

Bioluminescence imaging was performed on days 3, 5, and 7.5 following the introduction of *luc ^+^* cells into the distal tail (corresponding to post-operative days 10, 12, and 17.5, respectively). In general, when compared to normals, the clearance of bioluminescent immunocytes was delayed in lymphedema, but remained unimpaired in the surgical sham controls. Figure 4 depicts a series of imaging experiments for a representative pair of lymphedema and normal control mice. Relative photon density, expressed as the percent of the observed value on day 1, was significantly greater in lymphedema than in the normals, both at day 3 and at day 7 post-injection ( Figure 4).

### Large-Scale Analysis of Cutaneous Gene Expression in Response to Lymphatic Vascular Insufficiency (Lymph Stasis)

**Figure 5:**
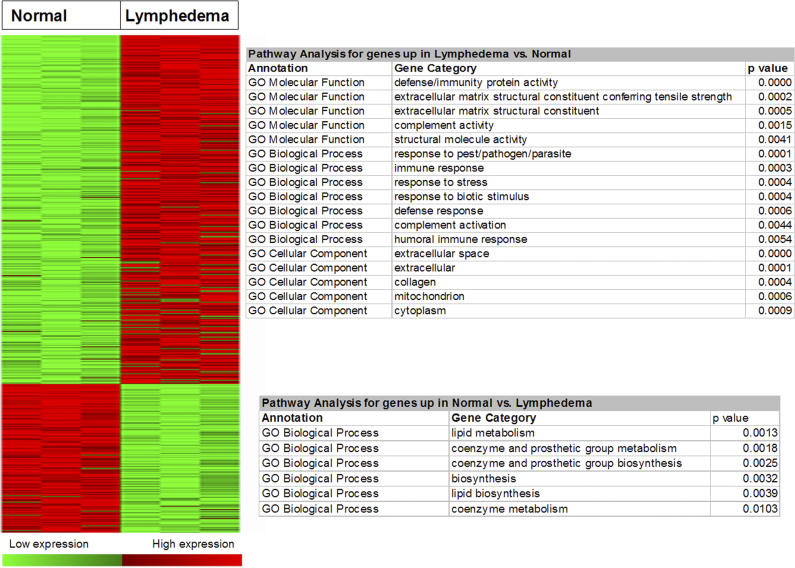
SAM Analysis of Microarray Data At an FDR of 5%, SAM analysis identified 429 upregulated genes in the lymphedema state versus 183 downregulated genes. There were no statistically significant differences between normal mice and surgical control animals (SAM, FDR < 25%). Enrichment analysis with the Fisher's exact test (EASE software) demonstrated several statistically significant ontologies.

cDNA microarrays containing a large portion of the mouse transcriptome were used to study the repertoire of genes expressed in the murine skin structures. Triplicate microarray experiments were performed using pooled RNA from the tail skin of female SKH-1 hairless mice representing three biological states: normal, lymphedematous, and surgical sham. Our analyses demonstrated significantly different patterns of gene expression in normal skin and the skin derived from lymphedematous mice. SAM, at a false detection rate (FDR) of 5%, identified 429 upregulated genes in the lymphedema state versus 183 downregulated genes ( Figure 5). There were no statistically significant differences between normal mice and surgical control animals (SAM, FDR < 25%). A complete list of differentially regulated genes is provided in Tables 3 and 4.

**Table 3:**
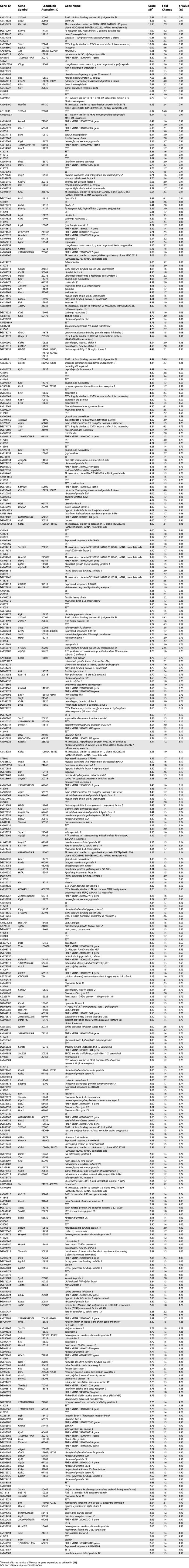
Upregulated Genes in Lymphedema versus Normal Control (SAM, FDR < 0.05)

**Table 4:**
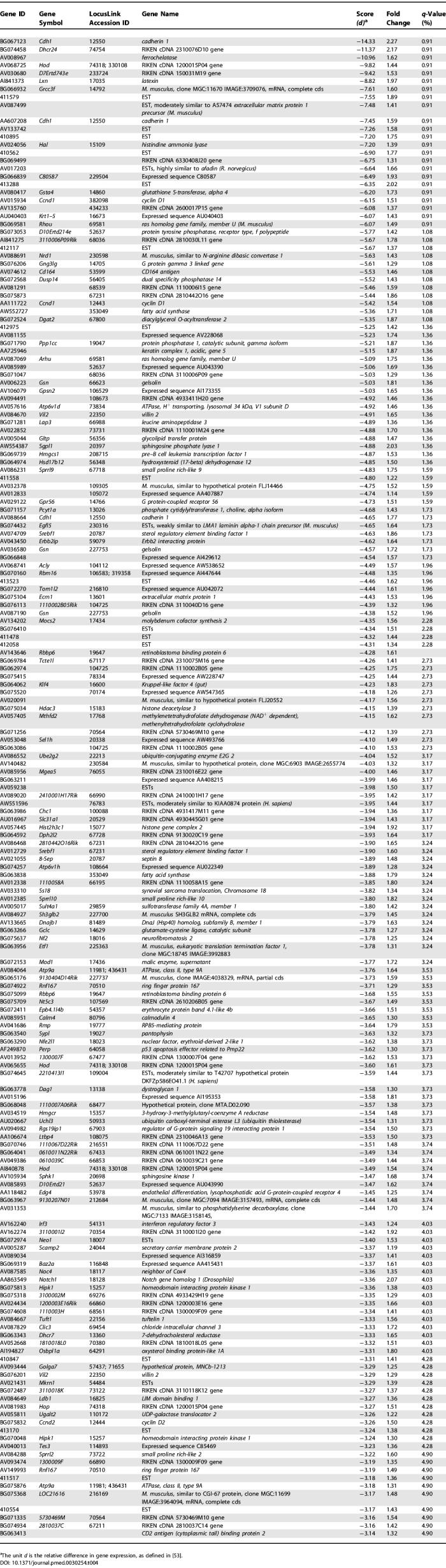
Downregulated Genes in Lymphedema versus Normal Control (SAM, FDR < 0.05)

To identify important biological themes represented by genes differentially expressed in the atherosclerotic lesions, we functionally annotated the genes using GO terms. Enrichment analysis with the Fisher's exact test (EASE software) demonstrated several statistically significant ontologies ( Figure 5; Tables 5 and 6), including several pathways associated with inflammation. The inflammatory processes, such as defense response, immune response, response to stress, response to pest/pathogen/parasite, and complement activation, represent both humoral immune response and innate immunity.

**Table 5:**
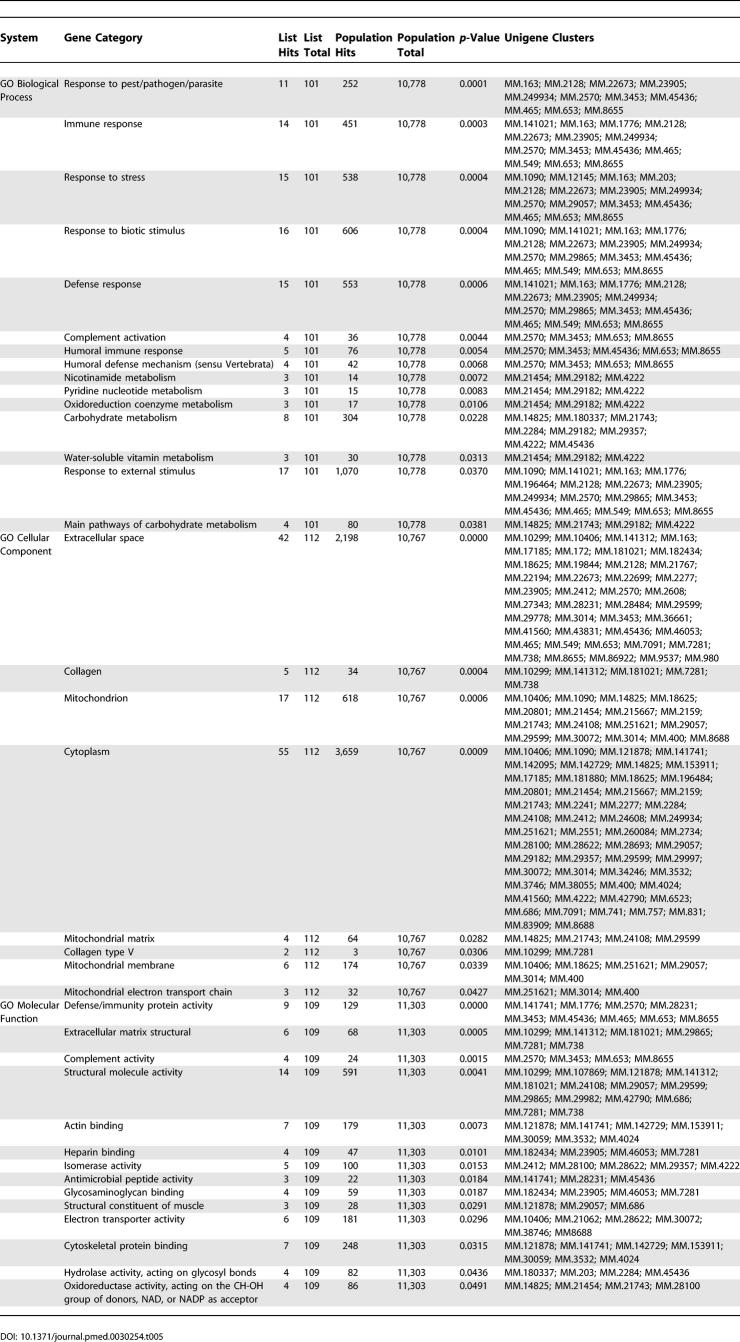
Pathway Analysis for Upregulated Genes in Lymphedema versus Normal Control (SAM, FDR < 0.05)

**Table 6:**
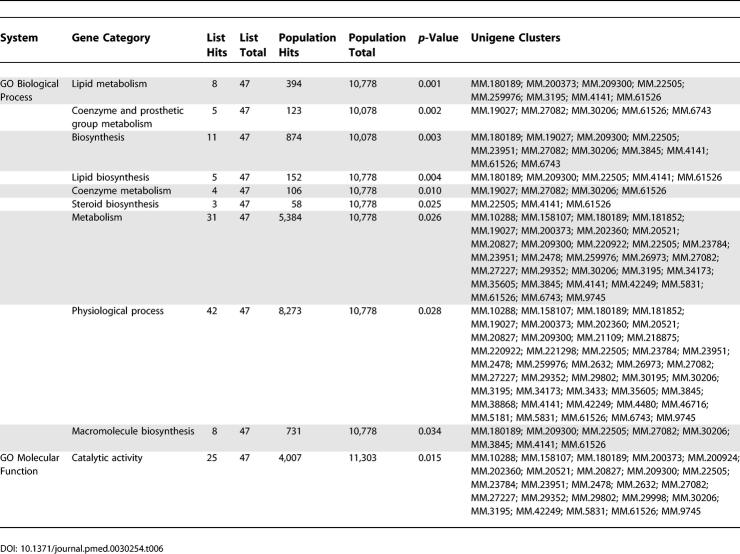
Pathway Analysis for Downregulated Genes in Lymphedema versus Normal Control (SAM, FDR < 0.05)

Further scrutiny of the list of genes whose expression is significantly altered in lymphedematous skin suggests that the disease process can be characterized by alterations within a relatively small set of functional attributes, as summarized in Table 7. These processes include acute inflammatory response, wound healing and fibrosis, angiogenesis, cytoskeletal organization, *Wnt* pathway activation, and adipogenesis.

**Table 7:**
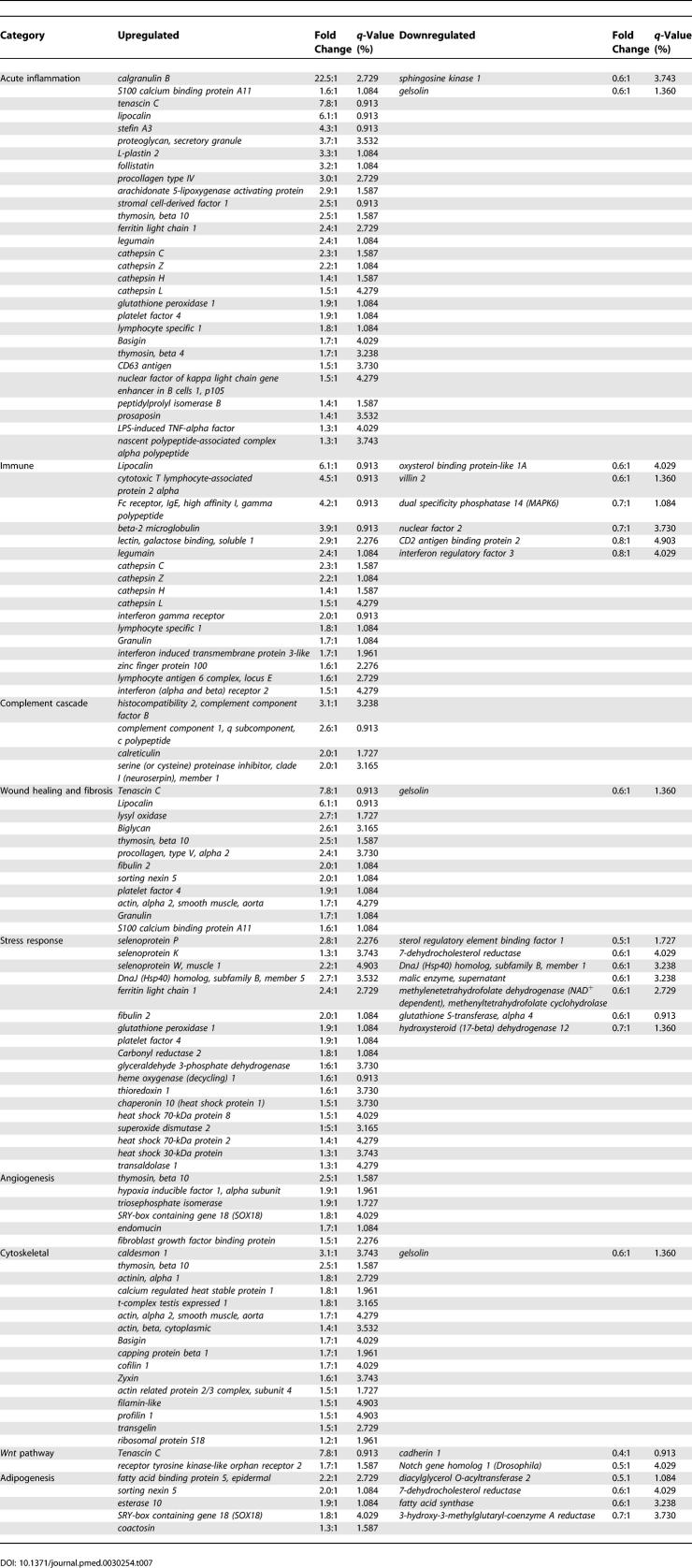
Functional Gene Expression Analysis in Experimental Lymphedema

### qRT-PCR Confirms the Accuracy of Microarray Hybridization Results

**Figure 6:**
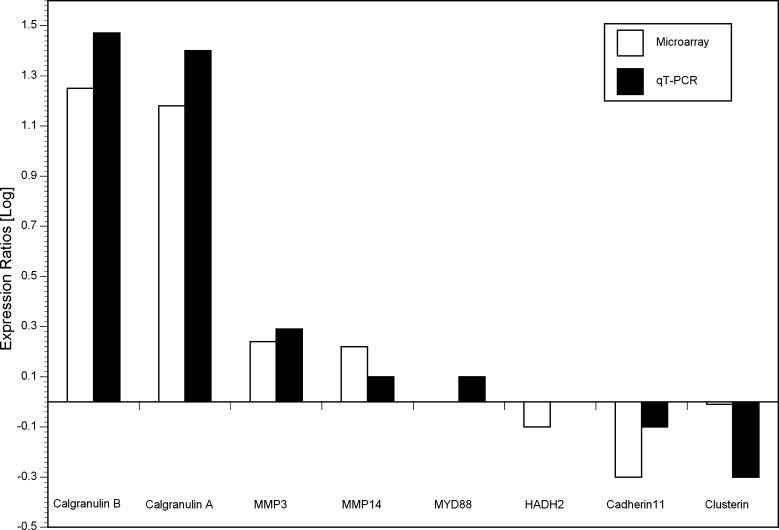
qRT-PCR Confirmation of the Results of Microarray Hybridization The graph represents fold-changes of expression in lymphedema, relative to normal controls, for each of eight representative genes, by microarray hybridization and qRT-PCR. For *MYD88* by microarray and *HADH2* by qRT-PCR, the log (gene expression) equaled zero. *HADH2, hydroxysteroid (17-beta) dehydrogenase; 2MMP, matrix metalloproteinase; MYD88, myeloid differentiation primary response gene 88.*

Differential expression of eight representative genes from various pathways was confirmed by qRT-PCR. The genes were selected to represent the spectrum of magnitude and direction of change of lymphedematous gene expression relative to normal. The genes assayed included *calgranulin A, calgranulin B, matrix metalloproteinase 3 (MMP3), matrix metalloproteinase 14 (MMP14), myeloid differentiation primary response gene 88 (MYD88), hydroxysteroid (17-beta) dehydrogenase 2 (HADH2), cadherin 11,* and *clusterin*. Overall, the results of the two methods correlated well ( Figure 6).

## Discussion

In this study, we have characterized a mouse model of lymphedema using in vivo functional imaging and histopathological correlation. This model of acute, acquired lymph stagnation closely simulates the volume response, histopathology, and lymphoscintigraphic characteristics of human acquired lymphedema. LYVE-1 immunohistochemistry demonstrates that this acute impairment of lymph transport is accompanied by an increase in the number and size of microlymphatic structures in the lymphedematous cutaneous tissues.

We have also undertaken molecular characterization of the disease process through comprehensive transcriptional profiling of the murine lymphedematous tail skin. We have identified a set of genes and molecular pathways that play a role in the unique biology of this cutaneous response to lymph stasis (lymphedema). Recognition of this molecular response pattern is likely to enhance our comprehension of the pathogenesis and biology of lymphedema.

The model has been elaborated to simulate the regional, acquired lymph stagnation that can arise after trauma, surgery, and cancer therapeutics [ 8]. Despite apparent rapid healing of the external cutaneous wound, the model features a stable, persistent edematous increase in the volume of the tail, accompanied by a profound inflammatory response; neither edema nor inflammation is seen in surgical controls.

The cutaneous inflammatory response observed in this model replicates clinical descriptions of human acquired lymphedema, where there is frequently evidence of concomitant chronic inflammation, and regional immune responses are distorted [ 2]. Architectural changes in the skin and subcutaneous tissues are often profound [ 3]. Chronic lymph stasis typically stimulates an increase in the number of fibroblasts, adipocytes, and keratinocytes in the skin. Mononuclear cells (chiefly macrophages) often demarcate the chronic inflammatory response [ 3]. In affected tissues, there is an increase in collagen deposition, accompanied by adipose and connective tissue overgrowth in the edematous regions [ 26].

In the current study, the molecular expression profile of lymphedema, observed in parallel with the histopathology and the dynamic immune traffic imaging, suggests that the deranged immune traffic plays at least a passive, if not an active, role in the pathogenesis of the disorder. In normal immune traffic, mononuclear phagocytes and lymphocytes from the tissues enter the afferent lymph vessels and the lymph nodes to elicit primary immune responses before reentering the vasculature [ 5]. It is conceivable that, in chronic lymphedema, the impairment of lymphocyte and Langerhans cell trafficking from skin to regional lymph nodes leads to inefficient clearance of foreign antigens, and provides the substrate for chronic inflammatory changes [ 4]. The complex biology of lymphedema is still quite poorly understood. Although it has been conjectured that inflammation may indeed trigger various forms of lymphangiogenesis [ 27, 28], the physiological sensors, signaling mechanisms, and cause-and-effect relationships that initiate post-natal lymphangiogenesis remain to be elucidated.

Transcriptional profiling has been utilized to identify genes activated in disease states and to refine targets for molecular therapy. Microarray technology has been applied to the elucidation of endothelial biology in health and disease [ 9, 29], as well as to the investigation of gene expression patterns in cutaneous diseases [ 30] and in a wide array of non-neoplastic diseases that entail inflammatory or immune responses [ 31]. This approach is particularly attractive for the problem of acquired lymphedema, where the heterogeneous cellular composition of the tissues exposed to lymph stagnation presupposes a very complex, interdependent pattern of gene expression. While the characteristic expression profiles of isolated lymphatic endothelia have previously been studied [ 6, 7], the current investigation represents the first in-depth molecular examination of the end-organ response to lymph stagnation. Despite the heterogeneous nature of the cellular material under investigation, the approach of high-throughput transcriptional profiling and statistical gene ontology analysis has disclosed discernable patterns of gene expression that appear to be representative of the disorder under scrutiny.

Transcriptional profiling can provide not only a gene-by-gene view of physiological alterations in a diseased state, but also a statistically rigorous identification of the biological processes that are induced or repressed in disease. This provides a much broader and more comprehensive view of the disease process as a whole than does a simple gene list, making generation of hypotheses about mechanisms more informed. Based on GO functional annotations for each gene on the array [ 32], we used Fisher's exact test statistical analysis to identify functional processes that are significantly induced and repressed in this disease model ( Table 7). The results of this analysis were quite interesting, illustrating that whole panels of genes involved in the immune response, stress response, and complement activation are induced in lymphedema when compared to controls. Among the most interesting of the upregulated genes involved in these processes are many encoding proteins that reflect the inflammatory process. Calgranulin B, highly upregulated in this experimental model, belongs to a family of small calcium-binding proteins that are highly expressed in neutrophil and monocyte cytosol. These molecules are found at high levels in the extracellular milieu during inflammatory conditions [ 33]. Calgranulins are potent stimulators of neutrophils and likely are involved in neutrophil migration to inflammatory sites. The levels of several of these proteins are markedly elevated in psoriasis, among other conditions [ 34]. Tenascin C is strongly induced by various pro- and anti-inflammatory cytokines, and its de novo expression is a reliable molecular marker for acute inflammation [ 35]. Peptidylprolyl isomerase B, also known as cyclophilin B, induces chemotaxis and integrin-mediated adhesion of T cells to the extracellular matrix in vitro [ 36]. Basigin is also involved in inflammatory processes and is proposed to be a receptor of cyclophilin A. Stromal cell-derived factor 1, also known as CXCL12, is a highly efficacious lymphocyte chemoattractant [ 37]. Platelet factor 4, also known as CXCL4, is a strong chemoattractant for neutrophils and fibroblasts. In addition to its putative role in inflammation, it has been implicated in the pathogenesis of atopic dermatitis. Upregulation of arachidonate 5-lipoxygenase activating protein suggests a role for leukotrienes in this acute inflammatory response; glutathione peroxidase may play an ancillary role. CD63 antigen can be interpreted as a marker of basophil activation and of degranulated neutrophils and monocytes. Legumain, an asparaginyl endopeptidase central to Class II major histocompatiblity complex presentation of microbial antigens, is a potential molecular marker of macrophage differentiation and function [ 38]. Follistatin is an activin antagonist implicated in wound repair; activin is an important participant in inflammation, repair, and cytoprotection in various organs, but its induction is restricted to certain types of inflammation and its release is dependent upon the inflammatory setting [ 39]. Nuclear factor kappa B is a transcription factor critical to the expression of a variety of chronic inflammatory disease states. The downregulation of gelsolin in this model is notable, inasmuch as hemostatic, inflammatory, and fibroblast responses are blunted in mice lacking gelsolin. Expression of nascent polypeptide-associated complex regulates formation of Fas-associated death domain (FADD) protein oligomers and modulates FADD-mediated signaling; FADD protein is a critical mediator of signal transduction pathways activated by several members of the tumor necrosis factor (TNF) receptor gene superfamily. Cathepsins are distinct intracellular acidic proteases that actively participate in the mechanism of antigen processing; conversely, the stefins are inhibitors of these cathepsins.

The immune response process is also statistically significantly induced in the lymphedema group versus controls. Proteins such as cytotoxic T lymphocyte-associated proteins are associated with activated T cell function and enhance TGF-β release by T cells [ 40]. Leukocyte (or lymphocyte) specific protein 1 (LSP1) is a multifunctional protein involved in the regulation of neutrophil motility, chemotaxis, adhesion, and B lymphocyte apoptosis mediated by membrane immunoglobulin M (mIgM) [ 41]. Beta-2 microglobulin is a major histocompatibility complex protein that presents peptide antigens on cell surfaces for recognition by T cell receptors. Lipocalin has recently been shown to participate in the response to bacterial growth [ 42]. Galactose binding lectin is a participant in the acute phase response [ 43]. Granulin is a high-molecular-weight secreted mitogen that is abundantly expressed in rapidly cycling epithelial cells and in the immune system [ 44]. High affinity Fc receptor for IgE is a key molecule in triggering the allergic reaction; it might be considered to be a mast-cell-specific protein. Interferon gamma has an important role in activating macrophages in host defenses.

One of the most interesting findings of our study is that lymphedema mirrors many of the mechanisms of the inflammatory state, in the absence of a potent inflammatory stimulus. One would not necessarily hypothesize, a priori, that this would be the case, since the only difference between lymphedematous animals and the controls (both normals and surgical shams) was the presence of acute, acquired lymphatic stasis, with no additional inflammatory stimulus; thus, lymphatic stasis induces many of the same mechanisms as inflammatory stimuli. This important observation is novel and has led us to pursue follow-up investigation intended to determine the efficacy of inhibitors of the inflammatory pathways in reversing the pathologic responses of the tissues that are seen in lymphedema.

The apparent absence of lymphatic markers in this transcriptional profile suggests that, in principle, the findings might not be specific for lymphedema. However, in parallel work performed in the same animal model, we have identified several lymphatic markers including, most importantly, upregulation of VEGF-C (but not VEGF-A) and VEGFR-3. Therapeutic induction of a lymphangiogenic response through administration of exogenous, recombinant human VEGF-C produces both an amelioration of edema and a downregulation of both of these markers of the lymphatic vascular response to acquired vascular insufficiency (unpublished data). Finally, as seen in Figure 3, LYVE-1 staining demonstrates specific lymphatic vascular changes in this model, including an increase in lymphatic microvascular size and density, in response to induced lymphatic vascular disruption. None of the morphological, histochemical, or gene expression changes referred to here are observed in animals that are subjected to surgery in the absence of induction of specific lymphatic injury (surgical controls), as described in this paper. Thus, it reasonable to state that the histological and molecular responses reflect lymph stagnation and not the more nonspecific responses of wound healing after surgical injury.

The cellular response to stress is another process that undergoes statistically significant induction during lymph stagnation. Among the stresses that can trigger this response are the elaboration of pathophysiological signals such as cytokines and eicosanoids [ 45]. The expression of a variety of heat shock proteins is upregulated in our model [ 45]. Additional evidence for the oxidative stress in lymphatic dysfunction is provided by the upregulation of heme oxygenase 1 (HO-1) [ 46]; it is a downstream effector of the potent anti-inflammatory interleukin IL-10 [ 47].

Upregulation of gene expression related to wound repair, and importantly, to fibrosis is also prominently seen. During wound repair granulin promotes granulation and neovascularization, and regulates inflammation [ 48]. The expression of fibulins is induced in the setting of injury, in response to various stimuli [ 49]. Biglycan (BGN) has been implicated in the regulation of matrix assembly, cellular adhesion, migration, and TGF-β activity [ 50]. Endoglin (CD 105) is a type III TGF-β1 receptor. It modulates the function of TGF-β1 by binding to and modulating signal transduction by the major type I and II TGF-β1 receptors. Lysyl oxidase plays a critical role in the biogenesis of connective tissue matrices. Alpha 2 actin has been identified as a marker of myofibroblast differentiation; all fibrocontractive diseases characterized by fibrosis entail the presence of myofibroblasts [ 51].

In addition to inflammatory/immune and stress responses, we have observed a gene expression profile that reflects alterations in the angiogenic response **.** Specifically, hypoxia inducible factor 1α has a key role in the cellular response to hypoxia, including the regulation of genes involved in energy metabolism, angiogenesis, and apoptosis. Alterations in the complement and *Wnt* pathways may also contribute significantly to the pathogenesis of the skin response to lymph stasis.

The observed differences between the lymphedematous animals and the surgical controls are noteworthy. In the absence of any observed delay in wound healing, overt infection, or inflammation, the gene expression profile in lymphedema, but not in surgical controls, is characterized by a remarkable induction of whole biological processes via coordinate upregulation of their component genes. These observations underscore the interpretation that lymphedema is a pathological process that is much more complex than a simple disorder of fluid homeostasis. Indeed, these gene expression profiles superficially resemble those of other recently elucidated inflammatory conditions, such as multiple sclerosis [ 31], psoriasis [ 52], and even atherosclerosis [ 53, 54].

The differentially expressed genes in our study likely arise from a number of different cell types, including not only the cellular components of the inflammatory response, but also other involved cell types such as keratinocytes, vascular endothelial and smooth muscle cells, and fibroblasts. We feel that it is important to study the gene expression of cells within the complex cellular milieu of the damaged end organ in lymphedema, because precisely the genes we are interested in are those whose transcription results from cell–cell or cell–interstitial fluid interactions. When one removes cells from the milieu, there are immediate changes in transcription that are not reflective of the disease state. We are, of course, interested in defining the cellular compartments that are expressing specific genes, and this topic is the focus of ongoing research in our laboratory that will be published in the future.

In summary, we have used an animal model of lymphedema that shares many clinical and histopathological features with human lymphedema to identify the biological processes and genes that underlie these features. The fact that inflammatory and immune processes are significantly induced suggests that these observations will provide a useful avenue for the investigation of novel pharmacologic strategies for lymphatic dysfunction [ 2]. This approach is particularly attractive in light of the observed parallels with other systemic inflammatory disease states for which effective therapies already exist. Ultimately, such therapies must successfully diminish the impact of the soft tissue fibrosis and adipose deposition that characterize the late disease [ 2]; in this regard, it is interesting to contemplate that expression of several such genes is detectably altered in this model, long before architectural evidence of the tissue abnormality is present. This identification of such genes provides an avenue for future investigation and, specifically, provides early insights into the elaboration of molecular therapeutics for this disease. Our purpose here has been to establish an animal model that can be used in two ways: first, to generate new hypotheses about unsuspected genes and pathways that are involved in lymphedema and, second, to eventually test potential pharmacologic and therapeutic interventions for their efficacy in vivo. We recognize that not all interventions that show promise in animal models translate effectively into treatments for human disease, but such in vivo testing is an essential prerequisite to human application and testing, and the establishment of a good, relatively inexpensive animal model is a very important step in allowing more high-throughput analysis of putative therapeutic interventions. These studies contribute to our understanding of the pathogenesis of lymphedema. Further characterization of the genes and molecular pathways identified through this effort will likely provide insights into potential novel strategies for molecular therapies.

## Supporting Information

Table S1 Annotated List of the cDNAs Included on the Mouse Transcriptome Microarray Utilized in These Investigations (75,633 KB MPG)

### Accession Numbers

The microarray data from these investigations have been deposited as dataset GSE4333 in the Gene Expression Omnibus database ( http://www.ncbi.nlm.nih.gov/geo). Accession numbers for the genes and proteins referenced in tables refer to the LocusLink database ( http://www.ncbi.nlm.nih.gov/projects/LocusLink).

## Abbreviations

dpm: disintegrations per minute
EST: expressed sequence tag
FDR: false detection rate
GO: Gene Ontology
*luc*: *luciferase*
qRT-PCR: quantitative real-time RT-PCR
SAM: significance analysis of microarrays
